# Assessing the governance environment for private sector engagement in health in Africa: Results from a multi-country survey

**DOI:** 10.7189/jogh.13.04113

**Published:** 2023-10-06

**Authors:** Michelle Amri, Omar Sam, Muriel Anye, Zandile Zibwowa, Humphrey Karamagi, Juliet Nabyonga-Orem

**Affiliations:** 1 World Health Organization Regional Office for Africa, Brazzaville, Republic of Congo; 2 Takemi Program in International Health, Harvard School of Public Health, Harvard University, Boston, Massachusetts, USA; 3 Centre for Health Professions Education, Faculty of Health Sciences, North-West University, Potchefstroom, South Africa

## Abstract

**Background:**

The role of the private sector in health is clear in many countries but engagement can be improved. The World Health Organization (WHO) developed a global strategy in 2020 focused on engaging the private sector in health service delivery through governance in mixed health systems and detailed six governance behaviours to guide its Member States. To operationalise these global ideas into practice, the Regional Office for Africa conducted a multi-country study to understand perceptions around the six governance behaviours. This article examines the perceptions of respondents from 13 African countries on the governance environment for private sector engagement in health.

**Methods:**

Data were collected through an online survey that was distributed to individuals from ministries of health and their partner organisations, private sector institutions and initiatives in countries and development organisations (n = 81) across 13 countries. The survey was based on the following six governance behaviours: build understanding, enable stakeholders, nurture trust, foster relations, align structures and deliver strategy.

**Results:**

Results showed that respondents had mixed perceptions of the governance environment for private sector engagement in health in their respective countries. Although 88% of respondents (n = 63/72) were familiar with the general inclusion of the private sector in national health sector plans, 63% of respondents (n = 45/71) noted there was limited or no integration of the private sector in the health system, and further, 28% of respondents noted there was no private sector reporting in health information systems (n = 19/69). Key opportunities presented in more than one governance behaviour include: (i) increasing private sector engagement in public sector activities, (ii) establishing clear roles and responsibilities through formal partnership agreements, (iii) improving data sharing through shared health information systems, (iv) incentives and subsidies, (v) capacity building, (vi) creating norms, guidelines, and regulations and (vii) conducting joint monitoring and evaluation. Many of these outlined overlapping concepts are not exclusive to one behaviour, thus, it is evident that when targeted, there is the potential to improve numerous governance behaviours. This further reiterates the view that the governance behaviours should be understood as connected and not unrelated areas.

**Conclusions:**

The study provides insight into the perceptions of respondents from select African countries on the governance environment for private sector engagement in health. These findings can inform the development of strategies and interventions to support and enhance private sector engagement in health in the region.

With increased attention paid to Universal Health Coverage (UHC) – understood as ensuring that “all people have access to the health services they need, when and where they need them, without financial hardship” [1] – and inclusion of UHC in national health strategies, attention paid to the practical realities of achieving UHC is needed. Given the collective challenges faced by the majority of governments in Africa, arguments have been raised that the public sector is unable to solely deliver the ambitious UHC agenda [2].

The private sector plays a large role in supporting health care, which can be seen through both service delivery and training. In terms of service delivery, a World Health Organization (WHO) cross-sectional survey in ten countries in the African Region found that the private sector delivered more health services in urban areas (55.9%) than the public sector or traditional health practitioners [3]. In Sub-Saharan Africa, 52% of outpatient care is provided by either the private for-profit sector or private providers [4]. It is also possible that the private sector is better equipped for service delivery, as surveys across more than 50 low- and middle-income countries (LMICs) found that 63% of private sector facilities reported having essential medicines, compared to only 38% of public facilities [4]. And in terms of training, of the 3894 health training institutes in the Region, 45.3% were owned by either private for-profit or private not-for-profit entities, compared to the remaining 54.6% owned by the public sector [3].

The response to the COVID-19 pandemic also accentuated the role of the private sector. For instance, the private sector in Kenya – guided by the government’s rapid response and preparedness plan, which the private sector was a part of – supported the COVID-19 response through supporting public awareness campaigns and laboratory testing [5]. In the case of Rwanda, the private sector was represented at the highest level, the command post level, which was established by the government of Rwanda to respond to COVID-19 [6]. In the Democratic Republic of Congo, Nigeria, Senegal and Uganda, the private sector strengthened laboratory, case management, risk communication, and health service continuity systems and supported increased access to COVID-19 testing services through partnerships with the public health sector [7]. However, despite private sector engagement (PSE) in health, there are challenges with varied approaches that are shrouded in mistrust [8].

To provide global guidance, the WHO developed a global strategy in 2020, *Engaging the private health service delivery sector through governance in mixed health systems: strategy report of the WHO Advisory Group on the Governance of the Private Sector for Universal Health Coverage* [2]. This strategy “seeks to redress a critical health system governance gap for the effective engagement of the private health service delivery sector in the context of Universal Health Coverage” [2]. The strategy seeks to build consensus about how to engage the private sector in health care service delivery and details six governance behaviours. These governance behaviours are believed to be “critical to private sector health service delivery governance” and are: (i) deliver strategy, “agreed sense of direction and articulation of roles and responsibilities”; (ii) align structures, “organizational structures to align with policy objectives”; (iii) enable stakeholders, “institutional framework that empowers actors”; (iv) build understanding, “collection and analysis of data to align priorities for action”; (v) foster relations, “working together to achieve shared objectives in a new way of doing business”; and (vi) nurture trust, “mutual trust amongst all actors as reliable participants” [2]. These governance behaviours are understood not to be sequential, but rather, work collectively to improve PSE in the health sector.

To operationalise these global ideas into practice, the WHO Regional Office for Africa (AFRO) convened the “multi-country workshop on PSE in the African Region” from 22-23 November 2022. Given that such multi-country consultations can lead to fruitful dialogue [9,10], the aim of the workshop was to respond to the demands of 17 countries that have included PSE as a priority theme in their 2022/23 biennial plans. Thus, the countries included in this workshop were: Angola, Botswana, Burkina Faso, Burundi, Cabo Verde, Chad, Comoros, Congo, Cote d’Ivoire, Kenya, Mauritania, Nigeria, Senegal, Sierra Leone, South Sudan, Uganda, and Zambia. In advance of the workshop, this study was conducted to understand participant perceptions around the aforementioned six governance behaviours. This article details the findings of this study to provide an assessment of the governance environment for PSE in the African Region.

## METHODS

### Study design

A mixed-methods survey was developed based on the WHO’s six governance behaviours to collect relevant data and the results section is structured accordingly. The survey was collaboratively developed across two regional offices of the WHO and validated by a team of experts at WHO headquarters who possess extensive experience working on PSE for health in LMICs. To reflect regional considerations and areas of interest, we added a few questions, such as around health equity, and conducted our own regional piloting both with internal staff and with counterparts working within governments across four countries. This led to adjusting questions to reflect in-country experiences and realities. The survey was sent by email in advance of the workshop to attendees, which included individuals from: ministries of health and their partner organisations, private sector institutions and initiatives in countries and development organisations. The link to the Qualtrics survey was sent in October and November of 2022 and the survey was available in English, French, and Portuguese. Responses in French and Portuguese were translated into English using computer software to facilitate analysis by the first author (MA). We recognise that machine-based translations may impact response meaning and tone but wanted to ensure perspectives from French- and Portuguese-speaking individuals was reflected in our analysis.

### Respondents

Key informants from the sectors of interest were identified by policy advisors working within WHO Country Offices. The survey was distributed to these 273 key individuals and responses from 81 respondents from across 13 countries were collected (response rate of 30%).

### Data analysis

For each of the governance behaviours, both closed- and open-ended questions were posed to respondents. For closed-ended questions, collected data are reported in the results section to indicate the number of respondents who noted the associated response. For the open-ended questions with varying response lengths, responses were compiled for each question and thematically analysed to consolidate responses to present a summary of results. Thematic analysis was selected as it aligned with our research goals of seeking to identify patterns across responses and gauge respondents’ views of the governance environment for PSE in health in Africa. From a more pragmatic lens, thematic analysis afforded a flexible method to analyse open-ended survey data and not report direct quotes to ensure respondents remain anonymous. More specifically, we conducted inductive analysis, meaning without a priori codes, to unveil recurrent respondent views. In addition to presenting recurrent themes in this way, unique or thought-provoking responses were also presented in grey text in Table S1 in the **Online Supplementary Document**, as we felt they can be considered by policymakers to provoke discussion.

## RESULTS

Data were collected from 81 respondents. The number of respondents per country, institutional affiliations of respondents and other demographic information is available in **Table 1**. Many respondents were development partners (n = 36/81; 44%) and public servants (n = 35/81; 43%).

**Table 1:** Respondent demographics

Characteristics	Number of respondents (n = 81) (%)
**Country**	
Angola	13 (16.05)
Burkina Faso	2 (2.47)
Burundi	11 (13.58)
Cabo Verde	1 (1.23)
Comoros	19 (23.46)
Congo	2 (2.47)
Côte d’Ivoire	8 (9.88)
Kenya	5 (6.17)
Mauritania	12 (14.81)
Nigeria	1 (1.23)
Senegal	3 (3.70)
Sierra Leone	1 (1.23)
South Sudan	3 (3.70)
**Health sector type**	
Development partner	36 (44.44)
United Nations agency	31 (86.11)
Other	5 (13.89)
Public sector	35 (43.21)
Private sector	10 (12.35)
Professional organisations (training and research institutes, association, councils, etc.)	4 (40.00)
Health service provider (for-profit)	3 (30.00)
Network organisation (federation, faith-based bureau, social franchise, etc.)	2 (20.00)
Health service provider (not-for-profit)	1 (10.00)

### Deliver strategy

For the first governance behaviour – delivery strategy – the question “does the national health policy (also known as a strategy in some countries) include the private health sector?” was asked. Set responses allow for gauging the nature of inclusion. From the 72 respondents who answered the question, there was an equal number of respondents who indicated that “the private sector is included in the national health policy but is vaguely referenced, meaning without specificity around roles” (n = 26/72; 36%) and that “the private sector is included in the national health policy with some specificity on entities and roles” (n = 26/72; 36%). Thus, signalling an opportunity to both explicitly mention the private sector and define roles more clearly. Coupled with the respondents who indicated that the private sector is “included in the national health policy or strategy with clear identification of entities and roles” (n = 11/72; 15%) – which was presented on the other end of the spectrum from the private sector not being mentioned (n = 1/72; 1%) – these responses collectively demonstrate there was general familiarity with the inclusion of the private sector in national health policies. However, there were some respondents also indicated they are unsure (n = 8/72; 11%), which perhaps means that there is a need for more awareness about this. From our assessment of 13 countries’ national health sector plans – reflecting the 13 countries respondents were from – we believe that the private sector is included in the national health policy with some specificity on entities and roles in all country plans (Table S2 in the **Online Supplementary Document** details select mentions of the private sector in country policies).

In asking “does the national health policy inform the work of the private sector?”, many respondents indicated that there was limited use of the national health policy (i.e. it is not reflected in operational plans) (n = 28/70; 40%) or that there is moderate use of the national health policy strategy (i.e. it is reflected in operational plans but has limited implementation) (n = 25/70; 36%). Few respondents indicated that the national health policy or strategy is not used to guide the private sector (n = 8/70; 11%) and even fewer indicated that there was demonstrated use of the national health policy or strategy (e.g. private sector operations reflect public sector roadmap and tools) (n = 8/70; 11%).

For the question, “is there an inclusive process for national health policy or strategy development and review?”, the same number of respondents indicated that the policy or strategy review includes selective participation of the private sector and civil society (n = 27/70; 39%) or that the policy or strategy review is inclusive of private sector entities and civil society (n = 27/70; 39%). Despite a few respondents indicating that their national policy or strategy review only includes the public sector (n = 8/70; 11%), that there is no inclusive policy or strategy review (n = 5/70; 7%), or simply that they were unsure (n = 3/70; 4%), the majority of respondents felt like their respective national policies have selective participation of private and civil society or are inclusive of private sector and civil society entities.

In response to “are there defined national health policy or strategy monitoring mechanisms in place that include the private sector?”, the overwhelming answer (n = 34/70; 49%) was that the monitoring mechanism is used in a limited way (e.g. at time of policy review, or only by the public sector). Remaining respondents indicated that the monitoring mechanism is defined, but there is no evidence of use (n = 12/70; 17%); there is no monitoring mechanism (n = 11/70; 16%); they were unsure (n = 8/70; 11%); or that the monitoring mechanism is used consistently (n = 5/70; 7%). Considering the combined number of respondents who either indicated that there is no evidence of use, that there was no mechanism, or they were unsure (n = 31/70; 44%), this emphasises the need to raise awareness of monitoring and evaluation frameworks which guide the performance assessment of health sectors in respective countries. Collectively, these responses signal an opportunity for both raising awareness of monitoring mechanisms and better including the private sector in this process.

And lastly, in asking “what do you think needs to be done to deliver a national health policy or strategy that is inclusive of the private sector?”, free-form responses were thematically synthesised and are presented in Table S1 in the **Online Supplementary Document**.

### Align structures

For the second governance behaviour – align structures – the question was asked “are private sector entities integrated into the health system?” The greatest number of respondents indicated that there was “limited integration of private sector entities in the health system (e.g. meaning larger urban health entities)” (n = 31/71; 44%). However, 18 respondents (25%) indicated that there was moderate integration (e.g. meaning a more diverse base of health entities) and eight respondents (11%) indicated that there was full integration of private entities in the health system – the latter of which is interesting when compared to the 14 respondents (20%) who indicated there is no evidence of integration in their respective countries.

In asking respondents “does your country have an essential health care package?”, the majority of respondents (n = 58/71; 82%) indicated that their respective country has an essential health care package, while six (8%) said no, and seven (10%) were unsure. Overall, it can be taken as a positive sign that most respondents are aware of the essential health care package, and that it exists in many of the respondents’ respective countries.

Only posing the question “do private sector entities deliver the services covered in the essential health care package?” to those who indicated that their respective country has an essential heath care package, resulted in many respondents indicating that the private sector partially delivers services (n = 34/58; 59%). Second highest, respondents noted that the private sector fully delivers services (n = 21/58; 36%). Thus, it appears there is a mix in the region of fully private provision and public and private provision.

In terms of whether guidelines are used to align public and private providers, many respondents noted that guidelines are available but inconsistently used (n = 34/71; 48%). However, a substantial number of respondents also noted that there are no guidelines available in their respective countries (n = 21/671; 30%).

And lastly, in asking “what do you think needs to be done to better align the public and private sectors and their actions?”, responses were thematically synthesised and are presented in Table S1 in the **Online Supplementary Document**.

### Enable stakeholders

Third – for enable stakeholders – many respondents indicated that there is limited private sector reporting in health information systems (n = 34/69; 49%) and several respondents indicated there is actually no private sector reporting in health information systems (n = 19/69; 28%). Combining the universal reporting (n = 2/69; 3%) and moderate reporting categories (n = 10/69; 14%), only 17% respondents selected these in total. Therefore, taken collectively, many respondents felt that there was relatively little engagement of the private sector in national health information systems in their respective countries.

In terms of other sources of private sector data being used (e.g. surveys, assessments, research), many respondents indicated that other data sources are partially used (n = 28/70; 40%). However, remaining responses varied, as 11 (16%) respondents indicated there are no other data sources available, nine (13%) indicated there are other sources available but are not used, 10 (14%) indicated there is evidence of triangulation of information sources and their use, and 12 (17%) were unsure. Therefore, it seems like there is an opportunity for increased use of private sector data.

And lastly, in asking “what do you think can be done to ensure gathering and sharing of information by both public and private stakeholders in an integrated manner?”, responses were thematically synthesised and are presented Table S1 in the **Online Supplementary Document**.

### Build understanding

Fourth – build understanding – in terms of whether the private sector engages the public sector, nearly half of the respondents (n = 31/69; 45%) indicated that the private sector does not engage the public sector in their respective countries. Only 19% of respondents (n = 13/69) indicated that the private sector regularly engages in dialogue with the public sector on areas of common interest. This signals an opportunity to not only think about how the public sector is engaging the private sector, but also reflecting on how the private sector considers the public sector.

Similarly, in terms of the public sector engaging the private sector, several respondents (n = 21/68; 31%) noted that there is no public sector organisation for PSE. However, when the remaining three response categories are considered collectively (excluding the “unsure” response), ranging from “the public sector has defined structures and processes for PSE” to “the public sector regularly engages in dialogues with the private sector on areas of common interest” about 56% of respondents felt this way about their respective countries (n = 40/68).

Many respondents noted there are no public-private coordination platforms (n = 24/68; 35%). Several respondents also indicated there are coordination platform(s) available but not formalised or used (n = 17/68; 25%) and that coordination platform(s) are formalised but used on an ad hoc basis (n = 15/68; 22%). Again, this perhaps signals an opportunity for either establishing coordination platforms or making better use of existing platforms.

And lastly, in asking “what do you think can be done to foster relations and build dialogue between public and private sector stakeholders?”, responses were thematically synthesised and are presented in Table S1 in the **Online Supplementary Document**.

### Foster relations

Fifth – foster relations – many respondents noted that regulations are in place in their respective countries but there are some gaps (n = 32/68; 47%). It is worth noting that only three respondents indicated there are absolutely no regulations in place (n = 3/68; 4%). Considering some of the responses to the earlier questions, coupled with the responses to this question, it seems the issue is not that regulations are absent, but rather that these regulations can be improved (whether in terms of reducing gaps or ensuring compliance with regulations).

In asking the question, “what public financing arrangements reach the private sector? Please select all that apply”, because respondents could select more than one answer, there were 135 selections. Training was selected by the most respondents, with 29 respondents noting that training reaches the private sector. About the same number of respondents selected access to commodities (n = 22), social health insurance (n = 22), and actually, that there are no public financing instruments for the private sector (n = 23). The latter is worth considering as a potential opportunity. The five respondents who selected “other” mentioned: the provision and distribution of vaccines from the government to private clinics (n = 2), patient exchanges (n = 1), the assignment of civil servants (n = 1), and a memorandum of understanding with a faith-based organisation (n = 1).

Most respondents felt like there is limited public sector capacity to ensure compliance (e.g. procedures in place but not fully implemented) (n = 30/67; 45%). When considering the limited (n = 30) and moderate capacity (n = 22) categories together, the majority of respondents selected these (n = 52/67; 78%). Only a few respondents indicated there is full public sector capacity to ensure compliance with regulations and rules (n = 3/67; 4%). The experience of these latter three respondents are worth further investigation, given that their views differ from the majority of respondents.

And lastly, in asking “what do you think can be done to enable stakeholders using regulation and financing towards national health goals?”, responses were thematically synthesised and are presented in Table S1 in the **Online Supplementary Document**.

### Nurture trust

And lastly, for the final governance behaviour – nurture trust – in asking respondents “are population and civic health interests the focus of public sector engagement with the private sector?”, it was clear that respondents were highly varied their views. These responses ranged from noting that these are broadly part of PSE (n = 16/67; 24%), to there is some specificity as part of PSE (e.g. some analysis of gaps) (n = 15/67; 22%), to these are not mentioned as part of PSE (n = 14/67; 21%), and these are specified as part of PSE (e.g. analysis considers gender, diversity, and equity) (n = 7/67; 10%). It is worth observing that a substantial number of respondents were unsure (n = 15/67; 22%), which may point to the need to stress the importance of population and civic interests in these public-private engagements.

In directly asking respondents, “is health equity a priority for you in your health sector work?”, an overwhelming number of respondents indicated that it is “always a priority” (n = 54/67; 81%). However, this was noted as not being a priority (n = 3/67; 4%) and sometimes a priority (n = 8/67; 12%) – and an additional couple of respondents were unsure (n = 2/67; 3%) – which draws attention to the need to reiterate the importance of health equity and ensure it is a shared goal across sectors.

Respondents also noted several challenges faced when working across sectors, which are presented in **Box 1**. However, in asking respondents “do measures exist to manage competing and conflictive sectoral interests?”, the responses were mixed. About the same number of respondents indicated that there are “no measures in place” (n = 16/66; 24%), “measures are in place but require pressure to prompt mitigation” (n = 17/66; 26%), and who were “unsure” (n = 17/66; 26%). There were also several who indicated that measures are in place but are not used for mitigation (n = 11/66; 17%). It appears that countries vary on measures to manage competing and conflictive sectoral interests.

Box 1 **Challenges faced working across sectors**

• Insufficient attention paid to health equity (including those with disabilities and rural residents), limited attention paid to improving equitable access to primary care and other services, and the general difficulty in effectively “achieving” health equity

• Lack of shared values or understanding (e.g. single health system, no spirit of teamwork, vested interests, corrupt practices) and inability to respect the conditions of each sector

• Weak integration and coordination across sectors

• Insufficient engagement of stakeholders in policy development and awareness of health issues (including the health budget)

• Little attention paid to accountability and/or compliance with legislation and regulations

• Practical or logistical issues (e.g. time management, difficult to perform, no coordination platform, data security)

• Limited political will and lack of funds (along with other generalisable issues that the health sector faces)

• Competing interests between the public and private sector (e.g. profit vs subsidising care and ensuring well-being)

When asking about the use of champions and/or brokers to engage other sectors, many respondents indicated that there are no champions or brokers (n = 30/63; 48%) and many were also unsure (n = 18/63; 29%). This signals an opportunity to draw on champions and/or brokers to enhance sectoral engagement. However, respondents who indicated that brokers and/or champions were used on an ad hoc basis with limited effect (n = 9/63; 14%), that brokers and/or champions were used more routinely to facilitate engagement (n = 4/63; 6%), or that brokers and/or champions were consistently engaged to facilitate the engagement and build trust across sectoral entities (n = 2/63; 3%), noted the various types of individuals and groups who typically play this knowledge broker and/or champion role, as outlined in **Box 2**.

Box 2 **Who typically plays this knowledge broker and/or champion role**

• Civil society and civil society networks

• Nongovernmental organisations

• Prominent academics

• Religious leaders

• Retired high-ranking civil servants/military officers

• Former Political Office Holders including Parliamentarians, Ministers, Governors, etc.

• Respected Traditional Rulers especially those that retired from high-ranking public/military offices

• Singers

• People affected by disease (e.g. HIV)

• The government (e.g. a specific department)

• Large international donors

• Professional associations

• Insurance companies (in a few cases)

Respondents were also mixed on their views about the sharing of resources, capacities, or skills for establishing trust between sectors. Most respondents indicated that there is ad hoc sharing of resources, capacities or skills (n = 22/65; 34%). However, at the same time, many indicated that there is “no sharing of resources, capacities, or skills” (n = 17/65; 26%). When considering these responses and that several respondents indicated they were unsure (n = 13/65; 20%), this presents an opportunity for increased resource sharing, capacities and skills between sectors.

And lastly, in asking “what do you think can be done to nurture trust between public and private health stakeholders and the populations they serve?”, responses were thematically synthesised and are presented in Table S1 in the **Online Supplementary Document**.

## DISCUSSION

The results of this study provide an overview of PSE in health in countries of the African Region. The valuable data collected can be thoughtfully considered to guide further studies, such as representative, focused, and tailored studies, and subsequently guide policy action in countries. For instance, our results demonstrate that 45% of respondents (n = 31/69) felt that the private sector does not engage the public sector, whereas 77% of respondents (n = 54/70) indicated that the public policy or strategy review includes selective participation or is inclusive of private sector entities and civil society. Investigating this finding could lead to the development of a harmonised monitoring system to provide a common platform for progress monitoring. When assessing the responses to the final question of each governance behaviour around what respondents believe is needed – presented in Table S1 in the **Online Supplementary Document** – there are several common responses across two or more of the six categories.

Key concepts that arose in more than one governance behaviour (**Table 2**) include: (i) increasing PSE in public sector activities – this concept appeared in the “deliver strategy”, “align structures”, and “enable stakeholders” governance behaviours, with suggestions to increase engagement through mechanisms for dialogue and consultation and involving the private sector in the preparation of national policy and strategic documents; (ii) establishing clear roles and responsibilities through formal partnership agreements – this concept appeared in the “deliver strategy”, “align structures”, “foster relations” and “nurture trust” governance behaviours, with the suggestion to draft a memorandum of understanding; (iii) improving data sharing through shared health information systems – this concept appeared in the “deliver strategy”, “align structures”, “enable stakeholders” and “build understanding” governance behaviours, with suggestions to include private sector data in health information systems and synchronising data collection and use between government and private stakeholders; (iv) incentives and subsidies – this concept appeared in the “align structures”, “enable stakeholders” and “foster relations” governance behaviours, with suggestions to provide incentives and subsidies for the private sector, such as for supply chain equipment or loan opportunities to invest in health, to encourage participation in health interventions; (v) capacity building – this concept appeared in the “align structures”, “build understanding”, and “nurture trust” governance behaviours, as capacity building of both private and public sector personnel was seen as one way to build trust and foster collaboration; (vi) creating norms, guidelines, and regulations – this concept also appeared in the “align structures” and “enable stakeholders” governance behaviours, which can entail developing pricing guidelines and creating an annual supervision schedule and executing it; and (vii) conducting joint monitoring and evaluation – this concept appeared in all governance behaviours with the exception of “foster relations”, as joint public-private evaluations and general inspections can help assess compliance with standards and national protocols.

**Table 2:** Concepts that came up in more than one governance behaviour category

Concept	Deliver strategy	Align structures	Enable stakeholders	Build understanding	Foster relations	Nurture trust
Increasing PSE in public sector activities	X	X	X			
Establishing clear roles and responsibilities through formal partnership agreements	X	X			X	X
Improving data sharing	X	X				
Incentives and subsidies		X	X		X	
Capacity building		X		X		X
Creating norms, guidelines, and regulations		X	X			
Conducting joint monitoring and evaluation	X	X	X	X		X

Many of these outlined overlapping concepts are not exclusive to one behaviour, thus, it is evident that when targeted, there is the perceived potential to improve numerous governance behaviours. This further reiterates the view that the governance behaviours should be understood as connected and not unrelated areas. Overall, there is a strong emphasis on enhancing collaboration and coordination between the public and private sectors to improve health outcomes. This includes increasing PSE, defining roles and responsibilities, improving data sharing, providing incentives and subsidies, and supporting capacity building efforts.

It is also worth observing how health equity came through in this study. In asking respondents if health equity is a priority, 81% indicated that it is always a priority. However, 4% of respondents expressed that this was not a priority, 12% indicated that it is sometimes a priority, and an additional 3% were unsure. Given that there is growing attention paid to health equity in global and public health [11-16], this finding draws attention to the need to reiterate the importance of health equity and ensure it is a shared goal across sectors. Coupled with the finding that a substantial number of respondents (22%) were unsure if population and civic health interests are the focus of public sector engagement with the private sector, this may point to the need to stress the importance of population and civic interests, alongside health equity, in these public-private engagements. Responses collected (as noted in Table S1 in the **Online Supplementary Document**) presented tangible opportunities for promoting health equity as a shared goal. For example, equity implications of the private sector's delivery of health services can be monitored using a tool that can be created for this purpose.

However, it is well established that working across sectors and ensuring shared goals is not easy. Respondents in this study raised several challenges faced when working across sectors, including practical or logistical issues, weak integration and coordination across sectors, insufficient attention paid to health equity, lack of shared values or understanding, and others as noted in **Box 1**. As a starting point to promote intersectoral and multisectoral action on health policy, evidence can be drawn on to improve chances of success [17-19] and creative solutions can be sought [20] – while ensuring that a focus on health equity is not lost in the process [21].

### Limitations

We see three limitations to this work around: (i) missing perspectives from select countries and health service providers; (ii) assessing perspectives vs behaviours and practices; and (iii) being unable to distinguish between cross-country differences. First, while the response rate was 30% and the perspectives of those in 13 countries were collected, the survey was unable to collect responses from respondents in Botswana, Chad, Uganda and Zambia. Therefore, exploring these perspectives in future studies is warranted. However, we feel it is important to note that this inquiry did not seek to provide a representative sample of views. Rather, our study explores regional perspectives to provide an important contribution that we believe is not diminished because it is not representative. Similarly, although there was a relatively low response rate from health service providers, it is important to note that their role in governance is limited. Thus, we do not anticipate this to have a significant impact on the results of this study. Second, the survey is designed to collect perspectives on the six governance behaviours. Although these perspectives provide a solid understanding of the governance landscape, we suggest further investigation into contrasting these perspectives with existing behaviours and practices (e.g. perspectives around PSE in national health strategies vs how national health strategies speak or engage the private sector). And lastly, this assessment provides a glimpse into the regional landscape for PSE governance, but countries will differ in unique ways. Thus, we recommend further investigation into understanding country-specific perspectives to provide tailored approaches.

## CONCLUSION

The recent actions taken by the WHO, including those in the African Region [2,3,22], should be capitalised on to continue building momentum in thoughtfully considering PSE in the delivery of health services, particularly in light of potential opportunities afforded through COVID-19 [23,24]. The results of this study provide an excellent starting point for identifying opportunities, such as through considering actions that are cross-cutting across governance behaviours (**Table 2**). However, these respondents’ views need to be balanced with the best interests of countries and should be considered alongside evidence. For instance, when asking respondents “what do you think needs to be done to deliver a national health policy or strategy that is inclusive of the private sector?”, the perspective to establish visible PSE partnerships (e.g. national committee) was apparent. However, great thought should be afforded to the establishment of such multisectoral committees, given the barriers they face in implementation [17].

## Additional material


Online Supplementary Document
