# Supplementary material for: Assessing the governance environment for private sector engagement in health in Africa: Results from a multi-country survey
Source: J Glob Health. 2023 Oct 6;13:04113. doi: 10.7189/jogh.13.04113 (PMC10556972; doi:10.7189/jogh.13.04113)
Supplement: Online Supplementary Document [file jogh-13-04113-s001.pdf]

Online Supplementary Document

Table S1. Respondents' views on what is needed for each governance behaviour[1]

Governance behaviour	Question asked	Responses
Deliver strategy	What do you think needs to be done to deliver a national health policy or strategy that is inclusive of the private sector?	• Establish visible partnerships (e.g., national committee) to increase engagement of the private sector, such as through formalizing mechanisms for dialogue and consultation (e.g., periodic meetings) and operationalizing existing mechanisms<br>• Better involve the private sector in the preparation of the national policy and other strategic documents, particularly from the beginning<br>• Define roles and responsibilities to enhance synergies (e.g., memorandum of understanding between the ministry of health and private sector)<br>• Draft relevant texts and establish monitoring mechanisms for PSE and performance (e.g., joint public-private evaluations and general inspections to assess compliance with standards and national protocols)<br>• Improve data sharing and the inclusion of private sector data in health information systems<br>• Affirm political will and commitment
Align structures	What do you think needs to be done to better align the public and private sectors and their actions?	• Integrate the private sector in public sector activities (e.g., joint monitoring committee; engagement in procurement; common planning, budgeting, and implementation; establish a unified health system; create an umbrella organization for private sector actors to enhance communication; capacity building of both private and public sector personnel)<br>• Develop norms, guidelines, and/or partnership agreements for PSE and each sector, such as a memorandum of understanding that outlines roles and obligations (e.g., can draw inspiration from other structures where alignment exists, establish regulations, develop pricing guidelines, create an annual supervision schedule and execute)<br>○ Ensure that these are disseminated to the private sector and that the public sector has the capacity to and will monitor and enforce private sector fulfilment of these guidelines<br>○ A first step can be to form a PSE technical working group and create a framework

[1] Please note: information in grey was not necessarily a recurring them but was included for consideration.

		• Provide incentives and subsidies for the private sector (e.g., for supply chain equipment and inputs)<br>• Health information systems: synchronize data collection and use between government and private stakeholders/providers<br>• Align the care package of private facilities with the national essential health care package<br>• Ministries of health can seek greater involvement from universities and health institutes<br>• Steer the attention of private health sector to have a greater focus on improving human health over profit<br>• Establish a tool to consider equity in health delivery for PSE<br>• Separate the supply and demand sides of care for PSE
Enable stakeholders	What do you think can be done to ensure gathering and sharing of information by both public and private stakeholders in an integrated manner?	• Strengthen the integration of the private sector in the collection, processing, dissemination, and use of health information (e.g., can begin by developing a policy and guiding principles, engage the federation of private health sector actors, draw on experiences of a malaria program)<br>• Harmonize reporting (e.g., information systems, indicators, electronic medical records) and integrate private sector data into health information systems (can be facilitated with a data sharing framework and a single, user-friendly, joint database)<br>• Ensure mandatory routine reporting and enforce reporting for relevant actors from all sectors<br>○ Consider incentives and sanctions for negligence and failures (e.g., government transfers can be based on services rendered as reported in the health information systems)<br>• Facilitate mechanisms for dialogue (e.g., regular discussion meetings, meetings to review data at various levels, PSE in ministry of health annual reviews) and capacity building (e.g., training on health information systems database)<br>• Engage different parties in data collection (e.g., patients, managers, community leaders, private sector representatives to secure private sector commitment)<br>• Collect disaggregated data for local use which may then promote the integration of private sector data in health information systems more generally<br>• Remind the private sector of the need to share data

		• Conduct a survey to identify private sector participation in the health sector[2]
Build understanding	What do you think can be done to foster relations and build dialogue between public and private sector stakeholders?	• Facilitate formalized dialogue between ministries of health and private sector actors, including civil society (e.g., host weekly meetings, establish a forum for networking, have a dedicated secretariat)<br>o Decentralize dialogue to countries<br>• Improve coordination and accountability (e.g., establish an exchange and/or coordination platform, joint planning, shared health information system platforms, create private-public network, transparency in the management of health care funds)<br>• Encourage participation in existing structures (e.g., private engagement in national health policies and strategies, private sector data in health reviews)<br>• Establish partnership agreements that have win-win-win solutions (e.g., formalize an inclusive and sustainable PSE or bipartite consultation framework)<br>• Develop a policy framework for PSE<br>• Use COVID-19 as an argument to reiterate that all sectors need to be engaged to better control epidemics and improve health (i.e., multisectorality)<br>• Reduce fragmentation between government entities that engage with the private sector and vice versa<br>• Consider that there should not be a sole representative for either the public or private sector, instead consider engaging with a multitude of corporatist organizations<br>• Explore the Global Fund's existing Country Coordination Mechanism to assess if other entities can join
Foster relations	What do you think can be done to enable stakeholders using regulation and financing towards national health goals?	• Improve the application of relevant government texts or create them where absent (e.g., health financing strategies that emphasize the notion of "invested with a public mission")<br>• Build regulatory capacity for institutions (e.g., audit training) or strengthen the functions of regulatory bodies using health laws<br>• Consider incentives for the private sector through financing mechanisms (e.g., loan opportunities to invest in health) and for regulatory bodies to enforce rules (also consider subsidies for both sectors)

[2] This is the goal of this study.

		• Develop clear regulations and a comprehensive regulatory framework and ensure compliance with standards<br>• Harmonize pricing at the private level and adopt strategic purchasing to ensure fair access to public and private providers<br>• Undertake classification of health services and products<br>• Improve the social insurance network<br>• Improve transparency (e.g., compulsory private sector disclosure for engaging in social insurance, re-establish government authority after criticism)<br>• Engage stakeholders with sustained communication efforts<br>• Promote a culture of excellence in the recruitment of health professionals and reinforce their capacities<br>• Monitor the implementation of policy decisions and equity implications<br>• Engage the national government to regulate public health facilities (instead of state governments)<br>• Fight against corruption and embezzlement<br>• Ensure goodwill in public-private interactions and foster a strong working relationship
Nurture trust	What do you think can be done to nurture trust between public and private health stakeholders and the populations they serve?	• Partnership, coordination, active participation, and communication<br>• Host regular multisectoral consultations to strengthen communication, improve coordination, and facilitate political and social dialogue<br>• Define the roles of each party and identify areas of collaboration<br>• Integrate and involve the private sector (e.g., can start by generating an investment case to attract the private sector, involve the private sector in developing government policy, engage the private sector in evaluation)<br>• Create an operational framework for experience sharing (sharing across countries) and joint initiatives<br>• Engage the community and community leaders in strategic decision-making and consider potential brokers/champions<br>• Develop public and private regulation (e.g., at the health facility level) and establish guidelines<br>• Fight corruption to avoid the misappropriate use of resources<br>• Engage in discussions of state budgets to improving financing for health

		• Initiate a value-based health care system to encourage the provision of quality services at an acceptable price<br>• Facilitate education on medical ethics to public and private health actors to lead to a convergence of common objectives

Table S2. Select mentions of the private sector in national health policies or health sector plans

Country	Title of national health policy or health sector plan<br><br>(years covered in the plan)[3]	Private sector mentioned? (yes or no)	Select mentions of the role(s) of the private sector[4]	"Does the national health policy (also known as a strategy in some countries) include the private health sector?"[5]		
				The private sector is not mentioned	The private sector is included in the national health policy but is vaguely referenced	The private sector is included in the national health policy or strategy with clear identification of entities and roles
Angola	Plano Nacional De Desenvolvimento Sanitário<br><br>(2012–2025)	Yes	"An effort to improve organization, accountability and the search for results will be required in order to create the foundations for sustainable health development. The PNDS foresees the strengthening of the sector's institutional framework, the search for forms of financing adapted to new needs, the dynamization of the policy for training cadres, the establishment of a fruitful relationship with the private health sector, and the active participation of local administrations, communities, and families in the process of improving health" (p. 4).<br><br>"The detailed budget of the programs will be presented in a second phase, following the discussion and validation at the national level of the proposed operational strategies and activities, through a broad			X

[3] The most recent plan that was available online was assessed.
[4] Reports in French and Portuguese were translated into English using DeepL software.
[5] This question was posed to respondents under "deliver strategy". We provide it here along with our assessment of each country's plan inclusion of the private sector that was agreed upon by M.A. and J.N.-O.

			public consultation of the document at the national, provincial and municipal levels, involving the different public and private actors and Civil Society for the harmonization and consolidation of the PNDS 2012–2025" (p. 21).<br><br>"Operational Strategies 1. integration of STI/HIV response initiatives across sectors (public, private and civil society)…" (p. 43).<br><br>"15. Define rules for the private sector to comply with protocols established in the country on the matter;…" (p. 80).<br><br>"The organization and management model of the National Health System should enable a sustainable improvement in the quality and performance of the universal system for Primary Health Care. The Angolan Executive, through the Ministry of Health, has defined the Municipal Health System as an integrating part of the National Health System, which should be understood as a set of services articulated among themselves and with other public and private sectors and society, which are coordinated by a governmental entity" (p. 158).<br><br>"The development of tertiary care, due to its national scope, should be based on complementarity between these institutions and other private initiatives. As recommended in the Long-Term Development Strategy, "Angola 2025", the tertiary network may be open to private initiative, becoming the fourth level of national priority, always in a Public-Private Partnership (PPP) regime, giving priority to those specialties where the greatest number of patients are evacuated abroad. Thus, the development of tertiary			

			hospital care institutions in the private sector should be stimulated. In this context, MINSA has been developing partnerships in specific situations, such as infant heart surgery and hemodialysis, with some privately managed public institutions, namely Girassol Hospital and Multiperfil. However, the private sector for tertiary and secondary care is not yet a complement to the SNS, due to poor regulation" (p. 178).<br><br>"Currently, there are difficulties in obtaining information about the initial training courses offered by private educational institutions in Angola, as well as the number of graduates from them. It is envisaged that the National Observatory of Human Resources, among other objectives, will coordinate with private institutions to collect this information in order to meet the human resources needs for the public sector" (p. 228).<br><br>"…The DNME has prepared and made available, for both public units and the private sector, Norms of Good Practice for the Storage and Distribution of Medicines and Health Products" (p. 277).<br><br>"Bodies and institutions responsible for implementation: 1. Implementing Agency: National Directorate of Medicines and Equipment, CECOMA; 2. Implementation support agencies of the Ministry of Health: CECOMA, Provincial Health Directorates, Municipal Health Departments, and the Private Sector (importers and distributors, pharmacies, herbalists, and laboratories licensed in the country); IGS: General Inspection of Health; General Secretariat of MINSA; INLS: National Institute for the Fight Against AIDS; DNSP: National Directorate of Public Health; GEPE:			

			Office of Studies, Planning, and Statistics of MINSA. CECOMA: Central Office for Procurement and Supply of Medicines and Medical Means; INSP: National Institute of Public Health and its National Laboratory; National Public Health Programs Saúde Pública; 3. National partners: Ministry of Higher Education; Ministry of Science and Technology; Public and private universities and higher institutes…" (p. 281).			
Burkina Faso	Plan National De Développement Sanitaire<br><br>(2011–2020)	Yes	"The national health system includes the public, private and traditional medicine and pharmacopoeia sub-sectors…Full communalization will have a major impact on the way the health system is managed at all levels. The private health care sub-sector has 358 facilities, most of which are for-profit (81.56%) and religious establishments located mainly in the cities of Ouagadougou and Bobo Dioulasso. Laws regulating the private practice of health care professions have been adopted" (p. 6).<br><br>"The private health sub-sector, which is developing without a master plan, is not sufficiently integrated into the health system and there are still gaps in the application of legal texts and in compliance with service delivery standards…However, there is (i) little development of sectoral dialogue between the government and its technical and financial partners, with insufficient functioning of the monitoring committee and technical commissions, (ii) no real coordination of projects and programs and their management units, and (iii) weak coordination at the level of the private sub-sector" (p, 7).<br><br>"In addition, the private health care sub-sector plays an important role in the provision of health care, particularly in urban areas. However, at the national			X

			level, private health establishments account for only 13% of the population’s health care needs, and the private sector’s participation in public health activities is low….<br>The number of Ministry of Health staff in the public sub-sector was estimated at 19,899 in May 2010. Private health care institutions employ nearly 1,800 people, 8% of whom are doctors (143), 37% nurses (669), 12% midwives and midwives (216), and the rest low-skilled personnel.8 The private health care sub-sector contributes to training with the medical school of the University of Geneva. The private health sub-sector contributes to the training offer with the Faculty of Medicine of the University of St. Thomas Aquinas and the private school Ste Edwige (training of paramedics) in Ouagadougou” (p. 9).<br><br>“The supply, distribution, availability and accessibility of health products have improved significantly in recent years. Drugs are imported by the Centrale d’achat de 10ormalizati essentiels génériques et des consommables médicaux (CAMEG) and nine (9) private wholesalers (Cophadis, Laborex, Pharmaplus, Faso Galien, Multi M, DPBF, ISDA, COPHARMED, Pharma International). As for distribution, in 2010, it is ensured by 144 private pharmaceutical dispensaries, 800 depots of essential generic drugs in public structures and 500 private drug depots” (p. 10).<br><br>“The implementation of the PNDS 2011–2020 will be done through three-year plans, hospital establishment projects, regional development plans and district health development plans. The latter will take into account communal development plans and the plans of all the other actors in the health area (private structures, NGOs/ associations, etc.)” (p. 37).			

Burundi	Politique Nationale De Sante<br><br>(2016–2025)	Yes	"ROLE OF THE PARTNERS: The various stakeholders in the health sector partnership (bilateral and multilateral partners, colleges, professional associations and learned societies, delegates of health professionals and trade unions, the private and faith-based sectors, traditional medicine, communities and civil society organizations, and organizations representing the sick) play an important role in the health sector" (p. 66).<br><br>"…In addition, in the absence of a national regulatory policy, some public sector physicians continue to work in the private sector. To address the shortage of doctors in rural hospitals, and in particular surgeons, a strategy for district hospital surgery has been developed" (p. 28).<br><br>"The number of paramedical schools increased from 4 in 2005 to 25 in 2013 (17 private, 2 denominational and 6 public). Over the same period, the number of medical schools producing general practitioners has increased from one to more than three53 without the same growth in teaching and supervisory staff or teaching materials. Compliance with standards by private training institutions is regularly a problem and raises questions about the quality of the staff they produce" (p. 28).<br><br>"However, recurrent stock-outs of essential medicines are observed, leading district pharmacies and health facilities to obtain supplies from private pharmacies in the absence of any documented competition and transparency mechanism" (pp. 26-27).<br><br>"Private sector and accredited: The Government facilitates public-private partnership to achieve the			X

			objectives set by the SNP. The collaboration is based on: (i) greater participation of the private and licensed sector in the provision of services to the entire population, (ii) better accessibility for this sector to the facilities offered by the Ministry of Health, (iii) better supervision and self-regulation of the sector, particularly in terms of health information and inventory of their members" (p. 68).<br><br>"(3) The peripheral level is represented by the health district, which is considered the basic operational entity of the national health system. It consists of an administrative base, a district hospital, and a network of public, accredited, or private health centers. At all levels of the health district, decisions are made collectively through health committees (COSAs) and management committees (COGEs) that serve as the interface between the health facilities and the grassroots communities in the health sector. The community plays a role in planning, implementing and monitoring primary health activities and finding appropriate solutions to local health problems and mobilizing resources. The peripheral level comprises 45 health districts" (p. 64)<br><br>"57. The private sector continues to develop without a formal master plan and is characterized by insufficient enforcement of legal texts and non-compliance with national norms and standards for the provision of health care and services. In addition, regulation and standardization in the sector, as well as collaboration and coordination within and between sectors, need to be strengthened" (p. 32).<br><br>"(1) The Partners for Health and Development Consultation Framework (PHDC) is the central			

			(strategic) body for steering, monitoring and evaluating the implementation of the NSP. Monitoring is done regularly during joint missions, during the RACs and through the thematic groups set up for this purpose. This is a multisectoral body made up of representatives of other development sectors working in synergy with the health sector (see Conceptual Framework – Chapter 5), TFPs, civil society and the private sector" (p. 70).<br><br>"…SIPHAR (Société Industrielle Pharmaceutique) is the only producer of (generic) medicines in Burundi and enjoys a de facto monopoly on the market" (p. 27).			
Cabo Verde	Política Nacional De Saúde<br><br>(2007–2020)	Yes	"VIII.1.2 – Strategies for the Private Health Sector: The guidelines for the organization and management of the private health sector, in recognition of its role as a partner in improving health care provision and as an integral part of the National Health Service, are directed towards: Preparation and publication of legislation to regulate the exercise of private activity in health, namely the functions, limits and articulation between the public and private subsystems; State inspection and supervision of private health care practice to ensure normal compliance with the established norms; Elaboration, in collaboration with professional associations, of deontological and ethical codes;…" (p. 33).<br><br>"4. Regulate, encourage, and supervise the development of the private health sector to participate fully in the provision of quality health care;" (p. 26).<br><br>"This guarantee also includes the defense of complementarity between the public and private sectors, with encouragement and support for the			X

			promotion of the private sector, philanthropic or for-profit, and its regulation, as a privileged partner in the task of making health care available" (p. 27).<br><br>"The private sector is an important element of the SNS and tends to assume its complementary role in the provision of health care. With legal support since 1989 [18], it developed, essentially in Praia and Mindelo, through the creation of medical and dental offices, clinical analysis laboratories, physiotherapy offices, and private pharmacies. Almost all professionals in the private health sector are in a regime of accumulation of work with public services. The State has not yet created the conditions to encourage the development of this sector or to exercise its regulatory and oversight role. Likewise, it has not provided itself with the necessary means to establish agreements capable of improving the use of available resources in order to achieve the desired complementarity between the public and private sectors. The lack of articulation between the two sectors with regard to a greater exchange of information is notorious, particularly in the sending of statistical data and in the notification of cases of diseases that must be reported. Given the increased expectations of Cape Verdeans and the dynamics of tourism, it is necessary that the private health sector develop and assert itself more and more as the desired complement to the public sector" (pp. 12-13).<br><br>"The partnership should also develop in a public-private framework, which is also useful for the health sector" (p. 49).<br><br>"Private health sector in sustained expansion, with domestic and foreign investment, playing a better role			

			as a complement to the public sector and offering a greater variety of services" (p. 20).			
Comoros	Plan National De Developpement Sanitaire<br><br>(2010–2014)	Yes	"In terms of health infrastructure, the Union of the Comoros has a National Referral Hospital (CHN) located in the capital of the country, two Regional Hospitals (CHR), one of which is in Anjouan and one in Moheli, and 17 District Health Centers (CSD), of which 7 are in Grande Comore, 7 in Anjouan and 3 in Moheli. Among these CSDs, there are two Medical-Surgical Centers (CMCs), including one in Anjouan and one in Grande Comore, and three Urban Medical Centers (UMCs), including one in each island, 52 health posts, including 26 in Grande Comore, 19 in Anjouan and 7 in Moheli, to which must be added 3 military health services, 4 CARITAS health centers and 15 medical offices and private clinics" (p. 25).<br><br>"The pharmaceutical sector is coordinated by the Directorate of Public and Private Establishments within the National Health Directorate, and essential drugs are supplied by the Comoros National Pharmacy (PNAC). Medicines are sold to the public by 17 pharmacies, 3 of which are run by PNAC and 15 by the private sector, as well as 59 private depots. All health facilities have a drug depot" (p. 27).<br><br>"Specific objectives: To increase the coverage of health infrastructures that meet the standards to 80%; To bring at least 50% of private facilities up to standard" (p. 47).			X
Congo	Politique Nationale De Sante<br><br>(2018–2030)	Yes	"Organization of the supply of health care and services: The health care supply system includes both the public and private sectors" (p. 13).<br><br>"After the liberalization decided in 1988, the private health sector developed with the growth of private			X

			establishments of all categories (polyclinics, clinics, medical centers and offices, pharmacies, private infirmaries). Also, the same agents working in the public sector can be found there, with the result that there are no health care personnel in the public structures. Most private health facilities are not licensed, a result of the poor regulation of the sector. This situation is compounded by the anarchic establishment of private health facilities" (p. 14).<br><br>"The formal private sector (notably insurance companies) has set up health insurance mechanisms" (p. 15).<br><br>"Drugs are distributed in the Congo through two channels. The public system supplies public health facilities and private non-profit health facilities. It is based on a central purchasing office for essential drugs. The private circuit supplies private for-profit health facilities. The unfortunate experiences of the Centrale Nationale d'Achats de Médicaments, vaccins et consommables médicaux essentiels (CENAMES) and the Congolaise de Médicaments Essentiels Génériques (COMEG), which were non-profit associations, led the government to create in 2016 a public establishment of an industrial and commercial nature: the Centrale d'achats des Médicaments Essentiels et des Produits de Santé (CAMEPS) for the procurement of essential drugs and health products for public and private non-profit health facilities. CAMEPS started its activities in August 2017" (p. 15).<br><br>"The private network has 195 facilities, including 12 clinics, 16 social medical centers and 50 medical offices" (p. 17).			

Côte d'Ivoire	Plan National De Developpement PND Résumé Synthétique[6]<br><br>2021–2025	Yes	"Through this new National Development Plan, which will mobilize approximately 59,000 billion CFA francs over the period 2021–2025, expected mainly from the private sector, we intend to accelerate the population's access to education, drinking water, electricity, health care, social protection, employment, etc. We will also pursue the development of the private sector, giving it a new lease on life, to make it the real engine of our economy. This will include the local processing of our raw materials, the strengthening of infrastructure and the promotion of national champions, in order to create more jobs, especially for young people and women" (p. 3).<br><br>"Private sector and investment development to improve the overall competitiveness of the economy. These include strategies and programs to promote the private sector and investment, develop finance and infrastructure, strengthen the business climate, and expand domestic, regional and international markets to increase trade and investment" (p. 15).<br><br>"The Plan also aims to transform the culture and practices within the administration and private institutions, to foster values and attitudes that are conducive to development. The aim is to raise the level of national and social awareness through a true cultural transformation, anchored in the educational system as well as in public administration and in private workplaces and places of worship, associations and non-governmental institutions" (p. 16).			X

[6] The summary report was assessed for inclusion of the private sector. However, there are likely to be more mentions in the three volumes that accompany the summary report.

			"Promotion of the Private Sector: Establishment of a new type of partnership with the private sector, with a modern, efficient administration at the service of the private sector; Valuation of entrepreneurship and its value in society; Promotion of national entrepreneurship; Reform of the institutional framework of the microfinance sector to improve the conditions of access to credit and ensure the protection of borrowers; Increased financing for entrepreneurs, SMEs and SMIs in the broad sense, through the strengthening of the system of financial guarantees and securities, and financial risk insurance services; Support for the interconnection of the Ivorian financial markets with the international and regional financial markets; Development of long-term contractual savings and mobilization of savings from public and private pension insurance systems; Mobilization of short-term household savings, particularly through Mobile Money operators; Development of new innovative financial products and services, expansion of the investor base and introduction of new financial players and; Strengthening financial inclusion through new banking solutions, including digital banks, and the correspondent banking system" (p. 17).			
Kenya	Kenya Health Policy<br><br>2014–2030	Yes	"The Kenya Health Policy 2014−2030 was developed under the stewardship of the national government over a period of two years through an evidence-based and extensive consultative process with stakeholders. These stakeholders included relevant government ministries, departments, and agencies; county governments; constitutional bodies; multilateral and bilateral development partners; and faith-based, private sector, civil society and implementing partners" (p. 7).			X

			"The health sector continues to be predominantly financed by private sector sources (including by households' out-of-pocket (OOP) spending). The private sector share of total health expenditure (THE) has decreased from a high of 54 per cent in 2001/02 (of which 44.8% constitutes OOP expenditure) to 37 per cent in 2009/10, (of which 24% constitutes OOP expenditure)" (pp. 12–13).<br><br>"2.4.5 Policy imperative: Create an enabling environment for increased private sector and community involvement in health services provision and finance: A framework for sector coordination and partnership was established in 2006 with the 19ormalization of the Kenya Health Sector-wide Approach (SWAp) process. Necessary instruments were defined, based on memoranda of understanding and a code of conduct to guide this dialogue and collaboration. In addition, the government supported health service delivery by non-state actors by providing access to public health commodities and medical supplies, and giving tax exemptions for donations in some of the facilities. The government also seconds critical public health staff to non-state facilities in specific cases, especially in under-served areas. However, the major beneficiaries of these initiatives have been faith-based service providers and not the private for-profit sector, which accounts for a 17.9% of all hospital admissions in 200714. Collaboration with private for-profit actors and alternative medicine practitioners is still weak. The government has also began providing health promotion and targeted disease prevention and curative services through community-based initiatives			

			as defined in the 2007 Comprehensive Community Health Strategy (MOH 2006)" (p. 18).<br><br>"The private health sector includes Private-Not-For-Profit (PNFP), Private Health Providers (PHP), Faith Based Organizations (FBO) and Traditional and Complementary Medicine Providers (TCMP), and collaboration will be strengthened through: development of a Public Private Partnership in Health(PPPH) policy framework; establishment of appropriate legislative frameworks and guidelines to facilitate and regulate the private sector in line with existing laws and regulations; and work with the private sector to reform incentive mechanisms (e.g. fiscal) that would attract registered private health practitioners to the under-served and difficult to reach areas" (p. 35).<br><br>"The primary care services will comprise all dispensaries, health centres, and maternity homes in both public and private sectors. Their capacity will be upgraded to ensure that all of them can provide appropriate services. It is envisaged that by the end of the policy period, the health centre will be the lowest level of a health facility…" (p. 41).			
Mauritania	Plan National De Developpement Sanitaire Volume 2[7]<br><br>(2017–2020)	Yes	"The challenge is also on the private side by regularizing the roles of pharmacies and pharmaceutical warehouses and guaranteeing the quality of imported products through single national markets, managed by CAMEC and involving importers, which will also have the advantage of increasing market levels and reducing intermediaries who tend to increase costs. The implementation of these actions within a framework of promoting the			X

[7] Only Volume 2 was assessed, as Volume 1 was unavailable for download.

			rational use of medicines and an integrated system (at least at the regional level) of management, supply, distribution and storage is also a real challenge for the coming years" (p. 9).<br><br>"Strengthened leadership and health governance through effective and efficient management of resources, effectively focused on results at all levels, and by strengthening the financing and participation of health system actors, particularly communities and the private sector" (p. 12).<br><br>"The SNIS will continue to receive special attention, enabling it to continue and strengthen its scaling-up efforts in order to effectively cover all public and private health structures. In this context, the initiatives undertaken (e.g. DIHS2) will be strengthened and put to good use for more effective monitoring of the health system and the implementation of the PNDS" (pp. 13–14).<br><br>"The monitoring of maternal and neonatal deaths has been made compulsory in all public and private health structures, and harmonized tools will be put in place to facilitate the annual publication of a national report on the audit of these deaths, which will be the subject of wide-ranging national meetings involving the governmental sectors, active nongovernmental organizations, the private health sector and the TFPs" (p. 17).			
Nigeria	Second National Strategic Health Development Plan<br><br>2018–2022	Yes	"Nigeria runs a pluralistic health care system with public and private sectors, modern and traditional systems providing health care" (p. 6).<br><br>"Nigeria has a growing private health sector which provides 60% of the health care services through 30%			X

			of the country's conventional health facilities – this includes not-for-profit services provided by faith-based and non-governmental organizations; and private-for-profit providers. The broader private health sector also includes traditional medicine providers, patent and proprietary medicine vendors (PPMVs), drug shops and complementary and alternative health practitioners" (p. 7).<br><br>"Routine health facility data collection is done through the DHIS 2 platform, which harvests data from 38,500 private and public primary and secondary facilities. While DHIS 2 average reporting rate in 2017 was 72%, timeliness remained low at 63%. Completeness is also a huge challenge with reported service data being significantly lower than national estimates from other known sources. There is very little to no reporting from the private sector despite the fact that the private sector provides 60% of healthcare services in the country" (p. 36).<br><br>"The private investment in health research and development is also very poor. Finally there is a disconnection between research in academic institutions in the country and the consumers of research findings. This calls for a pragmatic national research agenda that can inform public health policy and practice" (p. 37).<br><br>"Opportunities to broaden the revenue base for public health care financing including, sin taxes, aviation, VAT, health insurance, public-private partnerships e.g. corporate social responsibility, health impact bond and philanthropy have not been fully harnessed" (p. 38).			

			"In 2005, the FMOH developed the PPP Policy to provide a framework for the involvement of the private sector in the development of infrastructure and services in the country including health infrastructure and services. The FMOH PPP unit has facilitated these partnerships: The 'Warehouse in a Box' project (in Abuja and Lagos); The CAT-Lab and geriatric Wards in UCH; Installation of dialysis machines in FMC and Owerri; The LUTH/General Electric partnerships; Joint Venture Agreements between Crystal Thorpe Nigeria Ltd. and University of Abuja Teaching Hospital (UATH) for the management of the Trauma and Multi-specialist Centre at the Hospital and the concession of the Garki Hospital. Despite these few PPP successes, coordination and regulation of the private sector has remained weak" (p. 39).<br><br>"The private sector will be actively involved and engaged, through public-private-partnership (PPP) arrangements and relevant coordination and consultative mechanisms. The private sector actors, institutions and bodies, whether for profit or not-for-profit, working in the health system or providing healthcare services will be an integral part of NSHDP II implementation. The need to ensure an enabling and regulatory environment for meaningful private sector engagement is key to the success of NSHDP II" (p. 118).			
Senegal	Plan National de Développement Sanitaire et Social (PNDSS)<br><br>(2019–2028)	Yes	"As was the case during its development, the implementation of this PNDSS requires, once again, the involvement and commitment of all families of actors: administrative and territorial authorities, technical and financial partners, actors in the sector, social partners, private sector, civil society, communities, etc" (p. XIX).			X

			"…the evolution of the public and private supply of services and care through the option of densifying the health map in a multi-sectoral approach with the Emergency Community Development Plan (PUDC) and the Emergency Plan for the Modernization of Border Axes (PUMA)…" (p. 10).<br><br>"Private supply of health care services: It is significant. The mapping, carried out in 2017, counts 2,754 Private Health Structures (SPS) consisting of: 1,225 SPS of care including 03 hospitals, 37 health centers, 359 medical offices, 118 clinics, 443 paramedical offices, 132 company structures, 111 private health posts; 246 dental SPS including 207 dental offices; 33 diagnostic SPS including 26 biomedical analysis laboratories, 07 radiology and medical imaging centers; 1,250 pharmaceutical SPS, including 1,063 private pharmacies and 187 drug stores. In Dakar, the private medical service offer is 6 times the public offer" (p. 18).<br><br>"In addition to these aspects, it should be noted that the potential offered by the dynamism of the private sector is not sufficiently exploited and developed. In fact, this sector has nearly 17,000 permanent employees, including 1,592 specialist doctors, 582 general practitioners and 1,132 pharmacists… The private sector offers more than 30 medical specialties that are often not available in public facilities. The supply of health services from the private sector is not capitalized on to increase health coverage, nor is it sufficiently controlled by the technical authorities. The data and contributions of the private sector are insufficiently integrated into the calculation and analysis of indicators. Finally, the conditions of access to investment credits and insufficient tax incentives			

			limit the capacity of the private health sector to offer more affordable services to the general population" (p. 36).<br><br>"This chain comprises two circuits: (i) a public circuit in which the National Supply Pharmacy (PNA) mainly supplies the various public health structures; and (ii) a private circuit with private wholesalers who supply the pharmacies. The NPP, on the basis of an authorized list, also supplies wholesale distributors, partners, NGOs and religious structures" (p. 37).<br><br>"The private sector has a network of 1,063 private pharmacies and 187 drug depots. It thus offers a platform for the supply and distribution of specialty and generic products over most of the national territory" (p. 38).<br><br>"Strengthen the Public-Private Partnership:<br>Several strong measures are required to increase the contribution of the private sector, which is a privileged partner in the definition and implementation of health policies, in meeting the challenges facing the sector. In this perspective, the following measures should be considered (i) strengthening collaboration with private health sector organizations; (ii) developing and implementing a roadmap for improving the business environment in the health sector, particularly in terms of taxation and operating conditions; (iii) integrating the health sector business environment reform program into the agenda of the Presidential Investment Council; (iv) adoption of appropriate measures for the establishment of a legal framework conducive to the development of Public Private Partnership (PPP) projects in the health sector; and (v) improvement of the institutional			

			mechanism for the identification, planning and implementation of PPP projects within the MSAS" (p. 51).<br><br>"The State is the main source of funding for the PNDSS with 71% of total funding, followed by Technical and Financial Partners with 11%. The contribution of local authorities is 7%. Households contribute 6% of the total financing of the PNDSS, while the private non-health sector (RSE) contributes 5%" (p. 73).<br><br>"The private sector: It manages a large number of health facilities at different levels and contributes to the availability of care by offering quality services and qualified human resources. The development of the platform with the private sector has made it possible to strengthen the Public Private Partnership (PPP) and to make use of the comparative advantages of this sector" (p. 75).<br><br>"Data warehouse [(DHIS2 interoperable with other systems), integration of data from private and other sectors]. The data warehouse is a single database that groups or interoperates all databases. Today, the DHIS2 platform plays this role. This warehouse allows the sector to have a global view of all the data in the system and avoids fragmentation. All the data from the private health sector and other sectors involved in health and social action activities should be capitalized on in the sector's information system" (p. 81).<br><br>"The private sector, which is a new source of funding for the NSSP, contributes an overall rate of 5% to the financing of the sector. Moreover, the analysis shows			

			that this contribution increases over the years, from 4.2% in 2019 to 5.4% in 2028" (p. 87).			
Sierra Leone	National Health Sector Strategic Plan<br><br>(2017 – 2021)	Yes	"There is a considerable gap in knowledge and data on the private sector for healthcare in Sierra Leone. The private sector appears to be an important provider of services, especially a) in terms of health service provision at the secondary and tertiary level, with many hospitals known to be in operation across the country, and b) a considerable network of private sector pharmacies and informal drug peddlers. However, the sector is not formally organized around a series of trade associations, and data on utilization and sales either does not exist or is not easily accessible. Regardless of these gaps, the private sector will be considered as part of the overall approach to improving healthcare in Sierra Leone. Several new initiatives are already considering the role of private sector pharmacies, for example, including an effort to organize a network of providers who can provide drugs to Ebola survivors" (p. 30).<br><br>"Strategic Objective 9: Understand, engage and organize the private sector for healthcare:<br>9.1. Commission a private sector landscape analysis: The first step will be to understand the scope of private sector activities on healthcare in Sierra Leone. This will produce a clear overview and database of private sector hospitals, clinics, laboratory service providers and registered pharmacies, as well as some estimation of unregistered pharmacies and drug peddlers. Some comparison of public vs private sector utilization, service quality and cost will also be included as part of the exercise.<br>9.2. Develop a strategy to engage and organize the sector: Based on the landscape, the MoHS and its partners will come together for a series of discussions			X

			with key private sector stakeholders to discuss how the private sector can be better engaged in order to deliver improved health outcomes to the people of Sierra Leone. This may include the establishment of key bodies and associations – such as an association of private sector hospitals – that would help the public and private sectors engage in a more organized fashion. This should yield a private sector engagement plan that can be an addendum to the HSSP" (p. 30).<br><br>"To increase the number and proportion of new patients with TB (all forms) diagnosed and notified – (referred) by non-public service providers- (CHWs referral) and private health facilities from 24.1% in 2014 to 40 % by 2020" (p. 39).<br><br>"Health Workforce Education: At present, 25 public and private training institutions across Sierra Leone offer 56 different education programs, ranging from certificate to Master's level" (p. 49).<br><br>"Strategic Objective 4: Establish and promote partnerships among public, private and not-for-profit stakeholders: Under this strategic objective, MoHS aims to establish the cooperation and partnership from all sectors necessary to achieve the goal of this HRH Strategy. Across the government and regulatory agencies, inter-ministerial coordination is necessary to plan for, develop, and finance the national workforce. Within MoHS, effective, community-based participation is necessary to improve health worker accountability and management. Partnerships with private- sector, non-governmental, and faith-based organizations in service delivery, education, and technology is paramount to resourcing and implementing the HRH Strategy successfully" (p. 50).			

South Sudan	National Health Policy<br><br>(2016–2026)	Yes	“The Ministry of Health shall;<br>i. Institute measures for rational use of medicines and guide procurement and donations of medicines in public and private health facilities….” (p. 20).<br><br>“The Ministry of Health shall;<br>i) Advocate for increased private and public funding of the health budget.<br>ii) Ensure harmonization and alignment of development assistance for health funding with national policies, strategies and priorities<br>iii) Explore options for pre-payment schemes to hasten achievement of universal coverage where insurance options such as; private insurance, social insurance, insurance for specialised care, community health insurance etc. shall be considered.<br>iv) Introduce User Fees in private wings of public hospitals” (p. 21).<br><br>“Explore mechanisms for capturing private sector data through the Private Public Partnership for Health desk and health data from health facilities of partners, Military, Police, and Prisons” (p. 24).<br><br>“The ministry of health shall;… ii) Develop a public-private partnership for health policy to harness the complementary benefits of the private health sector.<br>iii) Strengthen partnerships with the private sector to include delegation, contracting, and collaboration among other forms of partnerships” (p. 27).			X
